# Seed germination characteristics of invasive ***Spartina alterniflora*** Loisel in Japan: implications for its effective management

**DOI:** 10.1038/s41598-020-58879-7

**Published:** 2020-02-07

**Authors:** Daisuke Hayasaka, Moe Nakagawa, Yu Maebara, Tomohiro Kurazono, Koya Hashimoto

**Affiliations:** 1 Faculty of Agriculture, KINDAI University, Nakamachi 3327-204, Nara, 631-8505 Japan; 2 Graduate School of Agriculture, KINDAI University, Nakamachi 3327-204, Nara, 631-8501 Japan; 3 Graduate School of Science, KOBE University, Rokkodai-cho 1-1, Nada-ku, Kobe, Hyogo, 657-8501 Japan; 4 Present Address: Department of Agriculture, Forestry and Fisheries, Mie prefectural office. Koumei-cho 13, Tsu, Mie 514-8570 Japan; 5 Present Address: NIPPON KOEI Co., Ltd. Kudankita 1-14-6, Chiyoda-ku, Tokyo, 102-8539 Japan; 6 Present Address: Civil Engineering and Eco-Technology Consultants Co., Ltd. Higashiikebukuro 2-23-2, Toshima-ku, Tokyo, 170-0013 Japan

**Keywords:** Invasive species, Invasive species, Plant ecology, Plant stress responses

## Abstract

*Spartina alterniflora*, intentionally or unintentionally introduced worldwide, has adversely impacted local Japanese ecosystems. Thus, prediction of future distributions of *S. alterniflora* and its management are required. Local population expansion after establishment depends heavily on asexual (clonal) reproduction, whereas sexual (seed) reproduction is one of the critical factors for estimating invasion success and the likelihood of colonization to new habitats. However, knowledge about the germination characteristics of *S. alterniflora* is lacking. Here, we report the environmental conditions suitable for germination of *S. alterniflora*, under variable conditions of cold stratification periods (0, 4, 8 weeks), temperature (constant, alternating temperature), light (light/dark, dark), and oxygen (aerobic, anaerobic). Cumulative germination rate of *S. alterniflora* increased with an increasing period of cold stratification. Its seeds clearly preferred aerobic conditions to germinate. Also, the germination rate was higher under alternating temperature than under constant temperature regardless of light and oxygen conditions in any cold stratification period. However, long-term cold stratification, alternating temperature, and aerobic conditions were more important for germination of *S. alterniflora* than light. Removal of soil seed banks within 8 weeks of cold stratification after seed dispersals with matured seeds may be effective approaches for disrupting the germination of *S. alterniflora*.

## Introduction

*Spartina alterniflora* Loisel (smooth cordgrass), native to North America and the Gulf Coast of the Mexico, is a perennial halophyte. This plant is common in saline or brackish water of the intertidal zone, usually occupying mudflats and sandflats with low to moderate wave energy^1^. Various biological traits of *S. alterniflora* such as fast growth, high tolerance to salt, and great reproductive capacity through both clonal growth and sexual reproduction, make this halophyte a good ecosystem engineer and a suitable species for ecological restoration^2,3^. For coastal erosion control, soil amelioration, and dike protection, *S. alterniflora* was intentionally introduced to the East Coast of the US, China, UK, and other regions^4–8^ from the West Coast (North Carolina, Georgia, and Florida) of the US. However, *S. alterniflora* escaped from the introduced areas due to its vigorous fecundity and then rapidly expanded their habitat range in most of the introduced areas^9,10^. For example, although the coverage of *S. alterniflora* introduced to China until 1985 was approximately 260 ha, it has increased more than 430 times (i.e., 112,000 ha) in just 15 years^8^. In addition, since *Spartina* species show significant adverse impacts on native coastal organisms, including plants, invertebrates, birds, and human-food molluscs, through competitive exclusion and habitat change^3,11,12^, these plants are listed among the 100 most hazardous invasive species in the world^13^. In Japan, *S. alterniflora* was first detected in Aichi Prefecture in 2008 and then in Kumamoto Prefecture in 2009^14,15^. Due to its rapid expansion in brackish waters and estuarine salt marshes of both Prefectures and the likelihood of ecological impacts on aquatic ecosystems, *S. alterniflora* has been declared an invasive alien species (IAS) on the Invasive Alien Species Act of Japan in 2014^16^.

For understanding of invasion success by introduced species to new habitats, it is beneficial to clarify the contribution of sexual (seed) and asexual (clonal) reproduction to their population growth^17,18^. Although some researchers reported that local population dynamics of higher plants after establishment depend heavily on asexual (clonal) reproduction regardless of native/non-native or invasive species^19,20^, sexual reproduction could also be important for colonization success to new habitats via pollen and/or seeds^17,21^. Furthermore, because the timing of the seed germination is critical for the survival of seedlings and their subsequent growth, species have evolved seed germination characteristics to adapt to particular habitat conditions^22^. It is well known that abiotic factors such as temperature, substrate, light, burial depth, and oxygen act as important signals to germinate adequately at “safe sites”^22–25^. Among these factors, alternating temperature, light, and aerobic conditions and cold stratification are particularly important triggers for the success of germination^22,26–31^. In addition, the germination rate of many hygrophytes and halophytes regardless of invasive and native species depends considerably on a combination of the environmental conditions mentioned above^32^. For these reasons, knowledge on the specific germination characteristics of a given species^22,33^ including *S. alterniflora* is essential for understanding germination success. Given that success of germination is the necessary condition for invasion success, germination characteristics can be regarded as one of the key predictors for evaluating the probability of invasion success by plants to new habitats^31,34^, because each species would germinate at a suitable time and habitat in accordance with present environmental conditions^22^. Therefore, if suitable environmental factors on the seed germination of *S. alterniflora* were clarified, we could predict potential habitats of the invasive plant and then lead to the planning of its effective management according to each habitat situation. Although there are some studies regarding the seed susceptibilities to salinity, sulphide, and alkali stresses^32,35^ and the dormancy release^1^, knowledge about the seed characteristics of the invasive *S. alterniflora*, especially on germination, is largely lacking.

Here, we investigated the suitable environmental conditions on seed germination of the invasive *S. alterniflora* in Japan, in particular the effects of cold stratification, temperature, light, and oxygen conditions which are important triggers for germination success. Additionally, based on our results, we discuss possible effective management strategies for invading populations of *S. alterniflora*.

## Results

**Figure 1:**
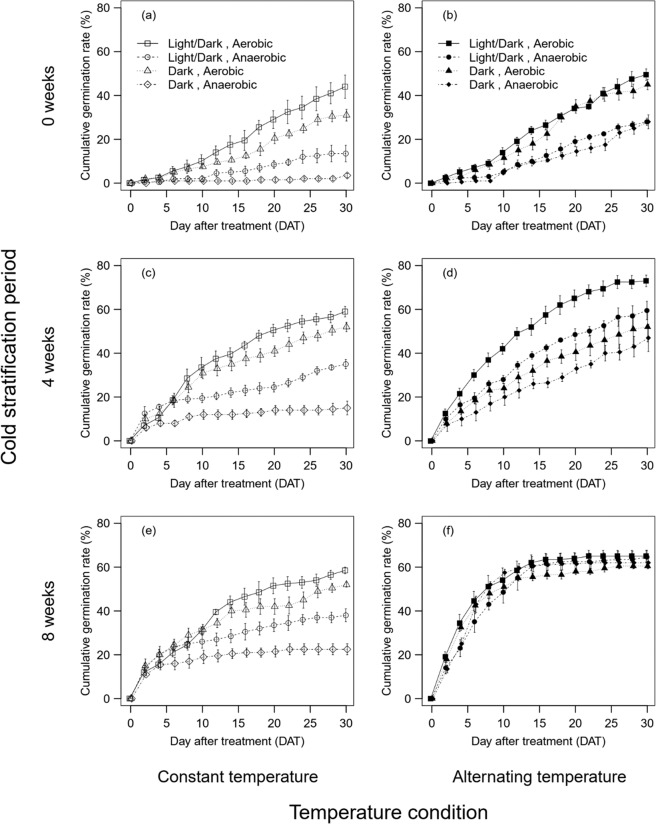
Differences in cumulative seed germination ratio (%) (mean ± S.E.) of *Spartina alterniflora* among the combination of the four environmental factors (i.e., cold stratification period (0 (**a,b**), 4 (**c,d**), 8 (**e,f**) weeks), temperature (constant (**a,c,e**) versus alternating (**b,d,f**) temperature), light (light/dark versus dark), and oxygen (aerobic versus anaerobic) conditions). Symbols is as follows: open square: constant temperature, light/dark and aerobic, open circle: constant temperature, light/dark and anaerobic, open triangle: constant temperature, dark and aerobic, open diamond: constant temperature, dark and anaerobic, solid square: alternating temperature, light/dark and aerobic, solid circle: alternating temperature, light/dark and anaerobic, solid triangle: alternating temperature, dark and aerobic, solid diamond: alternating temperature, dark and anaerobic.

**Figure 2:**
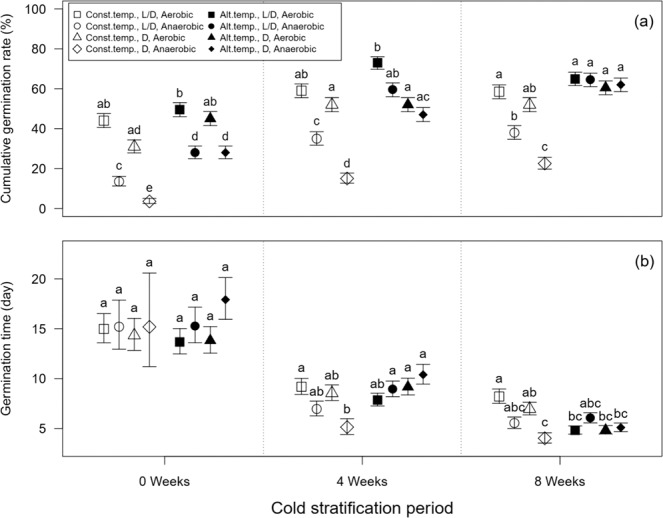
Comparisons of (**a**) cumulative germination rate (%) and (**b**) germination time (day) among different combinations of the four abiotic factors. Data of 30 days after starting the experiment were used. Back-transformed least square means ± S.E. were presented. Different letters indicate significant difference within the same cold stratification periods. Abbreviations were as follows: Const.temp., constant temperature; Alt.temp., alternating temperature; L/D, Light/Dark; D, Dark.

Although the seeds without cold stratification germinated to certain extent (maximum about 50%, Fig. 1a,b), the cumulative germination rate tended to increase up to approximately 60–70% with increasing length of the cold stratification (Figs. 1e,f, 2a). Also, different patterns on the slope of the seed germination were found with and without cold stratification (i.e., more rapid germination with increasing cold stratification periods) (Fig. 1). Germination rate under the alternating temperature treatment tended to be higher than that under the constant temperature treatment regardless of any other abiotic conditions (Figs. 1, 2a). Both dark and anaerobic conditions lowered cumulative germination rate, except for when cold stratification period was 8 weeks under alternating temperature condition (Figs. 1, 2a). Specifically, under constant temperature without cold stratification, the cumulative germination rate in light/dark and aerobic conditions (44%, the open square at 0 week of cold stratification period in Fig. 2a) was 13 times higher than that in the dark and anaerobic conditions (3.5%, the open diamond at 0 week of cold stratification period in Fig. 2a). In contrast, the differences in the rate between the two conditions (the solid square and solid diamond at 0 week of cold stratification period in Fig. 2a) were only less than twice under alternating temperature. Furthermore, differences in the germination rate between light/dark and aerobic versus dark and anaerobic conditions became smaller with increasing length of the cold stratification (Figs. 1, 2a). Especially, the cumulative germination rate among the treatments under alternating temperature were almost same (i.e., 60.5–65%) after 8 weeks of cold stratification (Fig. 2a). These disappearances of the negative effects of dark and anaerobic conditions were reflected in statistically significant interaction terms among temperature and cold stratification periods and the other two factors such as oxygen × temperature and light × cold stratification periods (Table 1).

**Table 1:** The effects of temperature, light, oxygen, and cold stratification periods on the cumulative germination rate (%) and germination time (day) of *Spartina alterniflora*. Cumilative germination rate was analyzed by a generalized linear model (GLM), whereas germination time was done by a linear mixed model (LMM). Bold shows statistical significance.

Explanatory variable	Cumulative germination rate			Germination time		
	*df*	LR-*χ*^2^	*P*	*df*	*F*	*P*
Temperature (T)	1	164.95	<**0.001**	1,69.2	0.75	0.39
Light (L)	1	52.69	<**0.001**	1,64.6	0.92	0.34
Oxygen (O)	1	188.44	<**0.001**	1,70.4	0.84	0.36
Cold stratification periods (CS)	2	211.49	<**0.001**	1,67.5	161.98	<**0.001**
T × L	1	5.36	**0.02**	1,72.7	5.42	**0.02**
T × O	1	66.36	<**0.001**	1,83.1	29.69	<**0.001**
T × CS	2	0.77	0.68	2,74.9	7.09	**0.002**
L × O	1	1.75	0.19	1,74.0	0.84	0.36
L × CS	2	6.46	**0.04**	2,69.3	1.79	0.17
O × CS	2	29.73	<**0.001**	2,79.0	1.38	0.26
T × L × O	1	12.59	<**0.001**	1,92.5	0.32	0.57
T × L × CS	2	7.52	**0.02**	2,79.5	0.56	0.58
T × O × CS	2	0.43	0.81	2,101.7	1.90	0.15
L × O × CS	2	0.18	0.91	2,84.3	0.73	0.48
T × L × O × CS	2	1.33	0.52	2,123.5	0.73	0.82

Similar to the results of cumulative germination rate, germination time was shortened with increasing cold stratification period (Fig. 2b). The effects of temperature treatment on germination time were not so obvious, but when cold stratification period was 8 weeks, alternating temperature condition tend to shorten germination time (Fig. 2b, temperature × cold stratification period, *F*_2,74.9_ = 7.09, *P* = 0.002, see Table 1). Unlike cumulative germination rate, under constant temperature condition, germination time tended to be shortened by dark and anaerobic conditions (Fig. 2b). This may have resulted from a decrease of germinating seeds in these treatments (Figs. 1, 2a), leading to a low fraction of slowly germinating seeds.

## Discussion

It is reported that the germination rate of *Spartina alterniflora* under constant temperature (25 °C), light (24 h-light), and aerobic conditions reaches to 80%^36^. In this study, maximum germination rate of the species under the light/dark and aerobic conditions without cold stratification was about 50% and less than 65–70% even after 4 or 8 weeks of cold stratification regardless of the temperature treatment (Fig. 1). The reason why the germination rate was relatively low in our study may be due to the short length of light treatment time (12-h per day) compared to a previous study (24-h per day)^36^, the seed status such as low proportion of matured seeds, and the timing of seed collection. Biber and Caldwell^1^ reported that storm surges, waves, and strong winds associated with hurricanes and the subsequent drought directly and indirectly influenced seed production of *S. alterniflora*. In June 2016, five months before the collection of seeds, flood and sediment disasters associated with a torrential rain occurred within a wide range of Kumamoto Prefecture, including the Oono River. Surely, most *S. alterniflora* populations were flooded and water-logged during this event, which happened during the growing season, thus disturbing seed production and/or maturation. The collection date of seeds can also affect the proportion of viable seeds available. A previous study suggested that the appropriate timing for collecting *S. alterniflora* seeds may be about 7 days after its seed production in Mississippi, USA^1^, although this study did not directly test the effects of collection timing on germination^1^. Thus, we do not have sufficient data about the relationships between seed viability and time after seed production to conclude that the timing of our seeds collection (i.e., about a month after seed production) was not appropriate.

In this study, cumulative germination rate of *S. alterniflora* under alternating temperatures was higher than that under constant temperatures, and light (light/dark) and aerobic conditions would act as triggers to increase its germination rate (Figs. 1, 2a). Situations with alternating temperature, light/dark, and aerobic conditions often occur on intertidal and supra-littoral zones due to tides. In general, it is assumed that for many higher plants including weeds and hygrophytes, the combination of these abiotic conditions to the proportion of seed germinating and dormancy release may be related to the possibility of detecting vegetation gaps, depth of burial, and water depth, which contribute to the detection of safe sites for germination^22,27,37,38^. Therefore, we assumed that *S. alterniflora* might relatively prefer intertidal and supra-littoral zones compered to subtidal zones where the invasive plant seeds submerge as its potential and suitable habitats for germination.

In general, seed germination of many plants regardless of native/non-native or invasive species is likely to be suppressed under constant temperature, dark, and anaerobic conditions as mentioned before^22,31^. However, in our study, higher germination rate and shorter germination time of *S. alterniflora* were found with long-term cold stratification even under constant temperature, dark, and anaerobic conditions (Figs. 1, 2a,b). Biber and Caldwell^1^ describe that caryopses of *S. alterniflora* require moist chilling for approximately six weeks to achieve high germination performance. Moreover, the effect of alternating temperature on the germination rate and germination time notably increased under the longest cold stratification period (i.e., 8 weeks), eliminating the negative effects of dark and anaerobic conditions (Fig. 2a). Therefore, we suggested that the combination of cold stratification and alternating temperature could synergistically facilitate the seed germinating of invasive *S. alterniflora*.

Based on our study, which indicates a high tolerance of *S. alterniflora* seeds to environmental stresses, after the seed dispersal with matured seeds, it would be effective to remove soils containing seed banks around *S. alterniflora* populations within 8 weeks of cold stratification, so as to foster inhibition of seed germination. Nevertheless, Sayce and Mumford Jr.^39^ reported that since the seed viability of *S. alterniflora* is short (roughly 8 months), this invasive plant does not maintain a persistent seed bank. In our study, we have only monitored the dynamics of *S. alterniflora* seed germination during 8 weeks. Based on a previous research^39^, the possibility of underestimating the seed germination rate (i.e., possibility of seed germinating after 8 weeks) cannot be denied due to the existence of dormancy seeds. Therefore, further study on the germination dynamics of *S. alterniflora* and considering the seed dormancy by longer-term monitoring above 8 weeks is needed. Also, further knowledge about the differences in seed-setting and germination rates between *S. alterniflora* and its competitors under the same environmental conditions is needed.

## Methods

### Seed collection

The sample collection was carried out following the method in Blum *et al*.^40^ considering intraspecies genetic variation with slight modification, which samples should be collected randomly from the colonies that are at least 5 m apart each other. In total, 200 *S. alterniflora* shoots with seeds were sampled from 8 population patches on intertidal mudflats of the Oono River of Uki city, Kumamoto Prefecture, Japan (32°37′53.8″ N and 130°39′35.7″ E) in late November 2016. This collection timing was about a month after seed production. The seeds were considered to be mature since all *S. alterniflora* seeds and shoots were brown at the sampling date. The collected seeds which are packed in zippered bags were brought back to our laboratory within that day, and then we have separated filled seeds (10,934 grains) from empty seeds (31,240 grains) by pushing the seeds with the thumb; the latter seeds were discarded. A previous study reported that although *S. alterniflora* appears to produce a large number of seeds (e.g., approximately 175,000 florets per pound^41^), most spikelets are empty, or contain a damaged or sterile caryopsis^1^.

### Germination experiment

To clarify the suitable environmental conditions on seed germination of *S. alterniflora* in Japan, we conducted an experiment examining the role of periods of cold stratification for the dormancy release, and the effects of temperature, light, and oxygen on seed germination. The experiment was conducted under fully-crossed combinations of the four abiotic factors (in total 24 treatments of four replicates (petri dishes) each) following Kato and Kadono^31^ with slight modifications. In addition, the various different combination among these abiotic factors is occurred on each location of estuaries and tidal flats due to tides.

The seeds were kept in an amber bottle (250 ml) sealed by aluminum foil, and filled with distilled water at 4 °C in darkness all day long (i.e., cold stratification) in a temperature-controlled incubator (LH-30–8CT, NIPPON MEDICAL & CHEMICAL Instruments Co., Ltd., Osaka) for 0 (control), 4, and 8 weeks. Then, 50 seeds with four replicate each (total 200 seeds) per treatment were placed on filter paper moistened with a distilled water every other day in 90 mm Petri dishes in diameter in the incubator under two temperature conditions: constant temperature (20 °C, 12 h-dark/12 h-light) and alternating temperature (15 °C/25 °C in 12 h-dark/12 h-light). The seeds were also exposed to either total darkness (dark) or a half of day photoperiod (light/dark) for testing the effects of light conditions to germination. Light was provided by white fluorescent tubes, producing a light intensity of 15 μmol m^-2^ s^-2^. Dark conditions were achieved by keeping the Petri dishes covered with aluminum foil. Counting the number of germinated seeds was done every other day under dim green safe light, which was obtained from 40 W fluorescent tubes (NEC FLR 40 SW/M; NEC Corporation, Tokyo) wrapped with green plastic films (110802; Toyo Corporation, Tokyo) following Toyomasu *et al*.^42^. For oxygen conditions, two treatments were used: aerobic, with seeds in Petri dishes exposed to the air via no closing their lids and anaerobic. The anaerobic conditions were achieved by sealing all Petri dishes in bags with Anaeropack – Anaero sachets and anaerobic indicators (MITSUBISHI GAS CHEMICAL Company, Inc., Tokyo). The Petri dishes kept in such anaerobic conditions were checked regularly (see below) after taking out of the sealing bags for a few seconds. Also, the sachets were replaced as needed to maintain anaerobic conditions.

A seed was considered to germinate when the seed coat ruptured^43^ and then the radicle emerged from the seed coat^31^. The number of germinated seeds was counted every other day^32^ during the monitoring period for 30 days^22^ and then germinated seeds were removed from the Petri dishes. Based on these count data, we obtained cumulative germination rate as the fraction of the number of germinating seeds per dish on each day, and germination time as the days elapsed until each seed germinated.

### Statistical analyses

To test effects of the four abiotic factors (cold stratification, temperature, light, and oxygen conditions) on cumulative germination rate of *S. alterniflora* 30 days after the starting the experiment, we used a generalized linear model (GLM) with the binominal distribution and a log link function. Cumulative seed germination rate was used as a response variable, and cold stratification (0, 4, and 8 weeks), temperature (constant versus alternating temperature), light (light/dark versus dark), and oxygen (aerobic versus anaerobic) conditions, and their interactions were used as explanatory variables. The effects of these abiotic factors on germination time were also analyzed by a linear mixed model (LMM). The ln-transformed germination time (day) of each seed was used as a response variable, and the four factors and their interactions were included as explanatory variables. Dish identify was included in the LMM as a random factor. The significance of each explanatory variable of the GLM and the LMM was tested by type-II likelihood ratio test and by type-II Wald *F* test using Kenward-Roger approximate denominator degree of freedom, respectively. These tests were followed by Tukey pairwise comparisons within the same cold stratification periods. These analyses were performed using the statistical software R. ver.3.4.2^44^.

## References

[1] Biber PD, Caldwell JD (2008). Seed germination and seedling survival of Spartina alterniflora Loisel. Am. J. Agric. Biol. Sci..

[2] Simenstad CA, Thom RM (1995). Spartina alterniflora (smooth cordgrass) as an invasive halophyte in Pacific Northwest estuaries. Hortus Northwest.

[3] Li B (2009). Spartina alterniflora invasions in the Yangtze River estuary, China: an overview of current status and ecosystem effects. Ecol. Eng..

[4] Spicher D, Josselyn M (1985). Spartina (Gramineae) in northern California: distribution and taxonomic notes. Madrono.

[5] Daehler CC, Strong DR (1996). Status, prediction and prevention of introduced cord grass Spartina spp. invasions in Pacific Estuaries USA. Biol. Conserv..

[6] Chung CH, Zhuo RZ, Xu GW (2004). Creation of Spartina plantations for reclaiming Dongtai, China, tidal flats and offshore sands. Ecol. Eng..

[7] Invasive Species Specialist Group (ISSG). Global invasive species database, http://www.issg.org/database/welcome/ (2005)

[8] An SQ (2007). Spartina invasion in China: implications for invasive species management and future research. Weed Res..

[9] Lacambra, C., Cutts, N., Allen, J., Burd, F. & Elliott, M. Spartina anglica: a review of its status, dynamics and management (English Nature, Northminster House, Peterborough PE1, 1VA, 2004).

[10] Morgan, V. H. & Sytsma, M. Alaska Spartina Prevention, Detection and Response Plan, https://www.fisheries.noaa.gov/resource/document/alaska-spartina-prevention-detection-and-response-plan (2010).

[11] Qian, Y. Q. & Ma, K. P. Bio-techniques, protection and wise uses of biodiversity. In Principles and Methods of Biodiversity Studies (eds. Qian, Y. Q. & Ma, K. P.) 217–224 (China’s Science and Technique Press, Beijing, China, 1994).

[12] Daehler, C. C. Spartina alteniflora Loisel, In Invasive plants of California’s Wildlands (eds. Bossard, C. C., Randall, J. M. & Hochovsky, M. C.) 296–299 (University of California Press, California, 2000).

[13] Lowe S, Browne M, Boudjelas S (2000). 100 of the world’s worst invasive alien species. Alien.

[14] Tamaoki, M. & Takizaki, Y. Current status and environmental effects of Spartina spp. in Japan. *J. Jap. Soc. Water Environ*. **38**, 61–66 (2015). (in Japanese)

[15] Kimura, T., Hanai, T., Kimura, S. & Fujioka, E. Identification of invasive alien species Spartina alterniflora in Japan using morphological characteristics as compared with native species Phragmites australis. *Jap. J. Benthol*. **70**, 91–94 (2016). (in Japanese with English abstract)

[16] Ministry of the Environment, Japan. List of regulated living organisms under the Invasive Alien Species Act, https://www.env.go.jp/nature/intro/2outline/files/siteisyu_list_e.pdf (2005).

[17] Weppler T, Stoll P, Stöcklin J (2006). The relative importance of sexual and clonal reproduction for population growth in the long‐lived alpine plant Geum reptans. J. Ecol..

[18] Liu H, Lin Z, Qi X, Zhang M, Yang H (2014). The relative importance of sexual and asexual reproduction in the spread of Spartina alterniflora using a spatially explicit individual-based model. Ecol. Res..

[19] Eriksson O (1992). Evolution of seed dispersal and recruitment in clonal plants. Oikos.

[20] Silvertown J, Franco M, Pisanty I, Mendoza A (1993). Comparative plant demography – relative importance of life-cycle components to the finite rate of increase in woody and herbaceous perennials. J. Ecol..

[21] Wilk JA, Kramer AT, Ashley MV (2009). High variation in clonal vs. sexual reproduction in populations of the wild strawberry, Fragaria virginiana (Rosaceae). Ann. bot..

[22] Baskin, C. C. & Baskin, J. M. Seeds: ecology, biogeography, and, evolution of dormancy and germination (Elsevier, 525 B Street, Suite 1900, San Diego, CA 92101–4495, USA, 2014).

[23] Harper JL, Clatworthy JN, McNaughton IH, Sagar GR (1961). The evolution of closely related species living in the same area. Evolution.

[24] Harper, J. L. Population Biology of Plants (Academic Press, London, 1977).

[25] Yin L, Zhang R, Xie Z, Wang C, Li W (2013). The effect of temperature, substrate, light, oxygen availability and burial depth on Ottelia alismoides seed germination. Aquat. Bot..

[26] Kawahara A, Takada H (1961). The germination of Trapella seed. I. Some factors influencing stimulation of germination. Indian J. Plant Physiol..

[27] Thompson K, Grime JP (1983). A comparative study of germination responses to diurnally-fluctuating temperatures. J. Appl. Ecol..

[28] Pons TL, Schröder HFJM (1986). Significance of temperature fluctuation and oxygen concentration for germination of the rice field weeds Fimbristylis littoralis and Scirpus juncoides. Oecologia.

[29] Yin L (2009). Cold stratification, light and high seed density enhance the germination of Ottelia alismoides. Aquat. Bot..

[30] Xiao C, Wang X, Xia J, Liu G (2010). The effects of temperature, water level and burial depth on seed germination of Myriophyllum spicatum and Potamogeton malaianus. Aquat. Bot..

[31] Kato R, Kadono Y (2011). Seed germination traits of Trapella sinensis (Trapellaceae), an endangered aquatic plant in Japan: Conservation implications. Aquat. Bot..

[32] Xiao Y, Sun J, Liu F, Xu T (2016). Effects of salinity and sulphide on seed germination of three coastal plants. Flora.

[33] Silvertown, J. W. Introduction to Plant Population Ecology, 2nd ed. (Longman, London, 1987).

[34] Elsey-Quirk T, Middleton BA, Proffitt CE (2009). Seed flotation and germination of salt marsh plants: the effects of stratification, salinity, and/or inundation regime. Aquat. Bot..

[35] Li R, Shi F, Fukuda K (2010). Interactive effects of salt and alkali stresses on seed germination, germination recovery, and seedling growth of a halophyte Spartina alterniflora (Poaceae). S. Afr. J. Bot..

[36] Callaway JC, Josselyn MN (1992). The introduction and spread of smooth cordgrass (Spartina alterniflora) in South San Francisco Bay. Estuaries.

[37] Benech-Arnold RL, Sánchez RA, Forcella F, Kruk BC, Ghersa CM (2000). Environmental control of dormancy in weed seed banks in soil. Field Crops Res..

[38] Vegis A (1964). Dormancy of higher plants. Annu. Rev. Physiol..

[39] Sayce, K. & Mumford, T. F. Jr. Identifying the Spartina species. In Spartina Workshop Record. (eds. Mumford Jr., T. F., Peyton, P., Sayce, J. R. & Harbell, S.) 9–14 (Washington Sea Grant Program, University of Washingron, Seattle, Washington, 1990).

[40] Blum MJ, Bandom KJ, Katz M, Strong DR (2007). Geographic structure, genetic diversity and source tracking of Spartina alterniflora. J. Biogeogr..

[41] Bush, T. Plant Fact Sheet: Smooth Cordgrass, Spartina alterniflora Loisel. (United States Department of Agriculture (USDA), Natural Resources Conservation Service (NRCS), Rose Lake Plant Materials Center, East Lansing, Michigan, 2002).

[42] Toyomasu T (1993). Light effects on endogenous levels of gibberellins in photoblastic lettuce seeds. J. Plant Growth Regul..

[43] Greenwood ME, MacFarlane GR (2006). Effects of salinity and temperature on the germination of Phragmites australis, Juncus kraussii, and Juncus acutus: implications for estuarine restoration initiatives. Wetlands.

[44] R Core Team. R: a language and environment for statistical computing, https://www.R-project.org/ (2017).

